# Impact of phospholipase C β1 in glioblastoma: a study on the main mechanisms of tumor aggressiveness

**DOI:** 10.1007/s00018-022-04198-1

**Published:** 2022-03-18

**Authors:** Stefano Ratti, Maria Vittoria Marvi, Sara Mongiorgi, Eric Owusu Obeng, Isabella Rusciano, Giulia Ramazzotti, Luca Morandi, Sofia Asioli, Matteo Zoli, Diego Mazzatenta, Pann-Ghill Suh, Lucia Manzoli, Lucio Cocco

**Affiliations:** 1 Cellular Signalling Laboratory, Department of Biomedical and Neuromotor Sciences (DIBINEM), University of Bologna, 40126 Bologna, Italy; 2 Functional and Molecular Neuroimaging Unit, IRCCS Istituto Delle Scienze Neurologiche Di Bologna, 40139 Bologna, Italy; 3 Department of Biomedical and Neuromotor Sciences, University of Bologna, 40126 Bologna, Italy; 4 Anatomic Pathology Unit, Azienda USL Di Bologna, 40124 Bologna, Italy; 5 Pituitary Unit, IRCCS Istituto Delle Scienze Neurologiche Di Bologna, 40139 Bologna, Italy; 6 Korea Brain Research Institute, Daegu, 41062 Korea; 7 School of Life Sciences, Ulsan National Institute of Science and Technology (UNIST), Ulsan, 689-798 Korea

**Keywords:** Phosphoinositides, Brain cancer, Glioma, Patients, Cellular signaling, Biomarkers

## Abstract

Glioblastoma represents the most lethal brain tumor in adults. Several studies have shown the key role of phospholipase C β1 (PLCβ1) in the regulation of many mechanisms within the central nervous system suggesting PLCβ1 as a novel signature gene in the molecular classification of high-grade gliomas. This study aims to determine the pathological impact of PLCβ1 in glioblastoma, confirming that PLCβ1 gene expression correlates with glioma’s grade, and it is lower in 50 glioblastoma samples compared to 20 healthy individuals. PLCβ1 silencing in cell lines and primary astrocytes, leads to increased cell migration and invasion, with the increment of mesenchymal transcription factors and markers, as Slug and N-Cadherin and metalloproteinases. Cell proliferation, through increased Ki-67 expression, and the main survival pathways, as β-catenin, ERK1/2 and Stat3 pathways, are also affected by PLCβ1 silencing. These data suggest a potential role of PLCβ1 in maintaining a normal or less aggressive glioma phenotype.

**Supplementary Information:**

The online version contains supplementary material available at 10.1007/s00018-022-04198-1.

## Introduction

Gliomas are primary brain tumors originating from the supporting neuroglial cells of the central nervous system (CNS) [1]. Gliomas can be classified into different histopathologic grades according to World Health Organization (WHO) grading system 2016 [2] and the grade is highly correlated with the prognosis of the patients [3]. Therefore, gliomas can be classified into low-grade gliomas (LGG), which display a more circumscribed growth pattern, and high-grade gliomas (HGG), which are mostly highly diffused and infiltrative, including Glioblastoma as the most common and lethal brain tumor in adults. Glioblastoma is a grade IV astrocytoma characterized by a heterogeneous population of cells that are genetically unstable, highly infiltrative, angiogenic and resistant to current therapies [4]. Glioblastoma can develop both de novo or evolve from a previous astrocytoma [5] and it is characterized by a very poor prognosis, with patients’ median survival around 12–14 months. The incidence of glioblastoma is about six cases/100’000 people/year and it often has a rapid progression of 2–3 months. Unfortunately, current glioblastoma treatment therapies are not effective in treating patients and only 3–5% survive up to 3 years or more [6]. Today, the main therapies for this kind of tumor are based on neurosurgical procedures, chemotherapy and radiotherapy, which are highly invasive and do not offer definitive cure. Glioblastoma histology displays necrosis and endothelial cell proliferation. Indeed, it has been demonstrated that glioblastoma possesses a high intra- and inter-individual heterogeneity which likely contributes to treatment failure and tumor recurrence [7]. Therefore, even the multi-therapeutic approach, tumor recurrence is common and part of it is due to the migration and invasion properties of the tumor cells, which invade the brain parenchyma, making complete removal of the tumor impossible with local or regional therapies [8]. For this reason, the study and the understanding of the pathological mechanisms that govern glioblastoma represents today a great challenge.

Several studies have shown the centrality of phosphoinositides (PIs) in the regulation of many mechanisms within the CNS and cancer [9]. PIs are lipid molecules [10] able to regulate numerous cellular processes, such as cell proliferation, survival, trafficking, chromatin remodeling and many other cell homeostasis pathways [11, 12]. Many types of PIs, that are implicated in both cytoplasmic and nuclear signaling, play a key role in many brain processes, from nervous system development to the modulation of synaptic plasticity [13, 14]. Among these enzymes, phosphoinositide-specific phospholipases C (PLCs), the most frequently studied phospholipases in cancer, are involved in several activities in the nerve cell, such as neuronal positioning and synaptic transmission [15]. PLCβ1, one of the most studied and highly expressed PLC isoforms in the CNS, have been pointed out as key enzyme and target in many brain processes [16], such as the control of endocannabinoid neuronal excitability [17] and the development of normal cortical circuitry [18]. Moreover, it was demonstrated that PLCβ1 signaling imbalance is linked to several brain disorders, including epilepsy [17, 19], behavior disorders as schizophrenia [20–23] and also neuro-oncological pathologies. Indeed, many pieces of evidence indicate that PI metabolism is implicated in the pathogenesis of glioblastoma, starting from the strong interactions between PLCs, and other mediators implicated in cell proliferation, differentiation, migration, and growth [24]. For instance, our recent publication reported the potential involvement of a specific PLC isoform, the PLCγ1, in the aggressiveness of glioblastoma. In particular, a positive correlation has been highlighted between its gene expression and tumor aggressiveness both in retrospective patients and in cellular models [25]. In addition, a study based on data analysis of the Cancer Genome Atlas (TCGA) and from Gene Expression Omnibus, revealed the potential role of PLCβ1 as biomarker in high-grade gliomas (HGG) [26]. In particular, an inverse correlation between PLCβ1 gene expression and glioma pathological grade has been highlighted, suggesting PLCβ1 as a potential prognostic factor and a potential novel signature gene for the classification of HGG. Moreover, it was shown in the same study a correlation between PLCβ1 expression and patient’s long-term survival, suggesting a correlation between PLCβ1 low expression and a worse prognosis [26]. Furthermore, PLCβ1 has been reported to participate to the migratory or metastatic potential of different cancer types, including breast cancer [27]. It was demonstrated that PLCβ1, through interaction with the Protein Tyrosine Phosphatase Receptor Type N2 (PTPRN2) protein, regulates cell migration in breast cancer cells by stimulating a decrease in plasma membrane levels of Phosphatidylinositol 4,5-bisphosphate (PtdIns(4,5)P2), an established PLCβ1 substrate which is known to control actin dynamics and cell migration [28]. Moreover, it was already evidenced the role of PLCβ1 as possible molecular marker able to define specific and personal therapeutic strategies in other tumor patients, as myelodysplastic syndromes (MDS) patients [29, 30].

Since the highly infiltrative and migratory abilities of glioblastoma cells into the healthy brain are responsible for glioblastoma malignancy and its worse prognosis, it is necessary to investigate the signaling pathways and the molecular processes that drive glioma cell motility and proliferation [31]. Glioblastoma cell migration and invasion are complex mechanisms regulated by different factors, that include the transition to a mesenchymal phenotype [32], through the activation of different transcriptional regulators as Slug [33], and changes in the extracellular matrix (ECM) [34] through matrix metalloproteinases (MMPs). MMPs expression in normal brain is low, but it increases in gliomas. More specifically, the metalloproteinases involved in gliomas’ invasive processes are MMP-2 and MMP-9, which correlate their expression with tumor grade and progression [35, 36]. Among the numerous signaling pathways involved in the mechanisms of migration, invasion and proliferation of glioma cells, one of the most studied is the β-catenin pathway, which belongs to the canonical Wnt-pathway, and it was demonstrated to be overexpressed in glioblastoma [37, 38]. Together with this pathway, also the mitogen-activated protein kinase (MAPK) pathway, which includes extracellular signal-regulated protein kinases 1 and 2 (ERK1/2), is often modulated in glioblastoma and implicated in invasive or proliferative tumor phenotypes [21, 39].

Glioma cell growth, differentiation and motility are regulated also by signal transducer and activator of transcription-3 (Stat3) pathway, which has been shown to be associated with glioblastoma oncogenesis and epithelial-mesenchymal transition (EMT) [40].

Considering all these studies, the understanding of the relation between the Inositide signaling and glioblastoma transformation could help to solve some of the missing key points of the physio-pathological mechanisms related to this tumor. The present work suggests, for the first time, that PLCβ1 and its downstream pathways could be involved in the aggressiveness of the tumor and could represent possible biomarkers for the molecular stratification of high-grade gliomas, correlating in silico data on glioblastoma patients, data on glioblastoma fresh-frozen samples and molecular data on cellular models engineered for PLCβ1 silencing.

## Materials and methods

### Data collection and tumor samples

The Chinese Glioma Genome Atlas (CGGA) RNA sequencing (RNA-seq) dataset (mRNAseq_325) with 325 glioma samples (103 WHO II gliomas, 79 WHO III gliomas, 139 WHO IV gliomas and 4 samples N/A of unknown nature) and corresponding PLCβ1 distribution and survival information, were downloaded from CGGA public database (http://www.cgga.org.cn/). The patients of validation set were divided into high- PLCβ1 and low-PLCβ1 expression group according to the cut-off value of PLCβ1 expression from CGGA dataset. Fifty glioblastoma tumor samples were obtained from the IRCCS Istituto delle Scienze Neurologiche di Bologna, Italy. This study was approved by the AUSL Bologna Ethical Committee (Comitato Etico di Area Vasta Emilia Centro della Regione Emilia-Romagna (CE-AVEC) N° 183/2019/OSS/AUSLBO evaluated on 20/03/2019) and informed consents were obtained from all participants.

### DNA mutation and methylation analysis

DNA from fresh/frozen tissues was extracted using the MasterPure™ DNA purification kit. Mutational analysis was performed using a next generation sequencing protocol evaluating the following 4 genes as indicated from WHO and cIMPACT-NOW guidelines [41]: isocitrate dehydrogenase 1 *(IDH1)* (exon 4), isocitrate dehydrogenase 2 *(IDH2)* (exon 4), histone *H3-3A* (exon 1), Telomerase Reverse Transcriptase *(TERT)* (promoter). Locus-specific amplicon libraries with tagged primers were generated using overhang adapters at 5′ based on Nextera™ sequence at the 5′ for Illumina sequencing; these adapters were recognized by a second round of PCR (eight cycles) to add Illumina P5/P7 adapters and sample-specific indices [42]. Amplicon products were purified with agencourt Ampure XP beads, quantified with the fluorometer Quantus™, pooled and loaded on MiSeq (Illumina). Each next generation sequencings (NGS) experiment was designed to allocate ≥ 1 k reads/region, to obtain a depth of coverage ≥ 1000×. FASTQ files were processed in a Galaxy Project environment [43], using hg38 human reference genome with BWA (Burrows-wheeler aligner), and visualized on IGV (Integrative genomics viewer). Only mutations with a variant allele frequency (VAF) threshold > 5% and a coverage depth of 10 × in both strands were reported. All mutations were checked in COSMIC database.

DNA methylation: bisulfite treatment of genomic DNA (50–500 ng) was performed using the EZ DNA methylation-lightning kit (Zymo Research Europe, Freiberg, Germany) according to the manufacturer’s protocol. DNA methylation was evaluated using targeted bisulfite NGS for O-6-Methylguanine-DNA Methyltransferase *(MGMT).* In brief, genomic sequences stored in the Ensembl genome browser (http://www.ensembl.org/index.html) were employed as query sequences to identify putative CpG islands in the promoter and the enhancer regions. MethPrimer (http://www.urogene.org/cgi-bin/methprimer/methprimer.cgi) designing was applied to identify CpGs and the best primers of choice. Libraries were generated using the same approach for mutation analysis with two PCR steps. FASTQ files were processed in a Galaxy Project environment following a pipeline described previously [44], in brief after filtering for quality > Q30 and for read lengths (> 80 bp), FASTQ files were then mapped by BWAmeth. BAM files were then in turn processed by MethylDackel using human GRCh38/hg38 as reference genome. The output files assigned the exact methylation level for each investigated CpG position [43]. In parallel, the web tool EPICT-TABSAT was used to confirm DNA methylation level results [45].

### Cell culture and lentiviral transduction

Human glioblastoma astrocytic cell lines U87-MG (HTB-14 ATCC, Old Town Manassas, Virginia, US) and U-251 MG (09063001 Sigma-Aldrich, St Louis, MO, US) were cultured in minimum essential medium eagle (MEM) (Corning, New York, US) with 10% FBS and 1% Penicillin/Streptomycin (Sigma-Aldrich) and Dulbecco’s Modification of Eagle’s Medium (DMEM) (Corning) with 5% FBS and 1% Penicillin/Streptomycin, respectively. Human embryonic kidney HEK 293 T cells (Genecopoeia Inc, US) were cultured in DMEM (Corning) with 10% FBS and 1% Penicillin/Streptomycin. Human Astrocytes HA, isolated from cerebral cortex (Catalog #1800, ScienCell Research Laboratories, California, US), were cultured in Astrocyte Medium (AM, Cat. #1801, ScienCell). All cells were maintained in a humidified incubator at 37 °C with 5% CO2.

HSH096803-LVRH1GP-b, HSH096803-LVRH1GP-c and CSHCTR001-LVRH1GP control vectors (Genecopoeia) were used to construct lentiviruses to silence PLCβ1 and express green fluorescent protein (GFP) as well as lentiviruses coding only for GFP, as control. The Lenti-Pac HIV expression packaging kit (Genecopoeia) was used as packaging system to transfect HEK293T cells according to manufacturer’s protocol. The supernatants containing the viruses were harvested 24–48 h after transfection and filtered through a 0.45 µm cellulose acetate filter. To perform viral transduction, U87-MG and U-251 MG were plated in a 6-well plate at a concentration of 5 × 10^5^ cells/well. Primary HA Astrocytes were seeded at a concentration of 5 × 10^4^/cm^2^ in 6-well and 12-well plates. The next day virus supernatants together with polybrene 8 µg/ml for U87-MG and 6.4 µg/ml for U-251 MG and HA, were added to the target cells.

U87-MG and U-251 MG cells were treated with 2 µg/ml and 1,5 µg/ml of puromycin (Sigma-Aldrich), respectively, 48 h after transduction and were left in culture for one month to obtain a fully selected clone with stable PLCβ1 silencing. Instead, HA primary astrocytes were treated with 1 µg/ml of puromycin 24 h after transduction and analysed after 96 h.

### RNA extraction, reverse transcription, and real-time PCR

RNeasy lipid tissue mini kit (Qiagen, Hilden, Germany) was used to extract total RNA from fresh-frozen clinical tissues following manufacturer’s protocol. Nanodrop spectrophotometer was used to quantify the extracted total RNA. Subsequently, Qubit fluorometer (Thermo Fisher Scientific, Waltham, MA, USA), and Qubit RNA IQ Assay, were used for quality analysis of the RNA extracted. Only samples with Qubit IQ number of seven or greater, were selected for further experiments. Total RNA extracted from different brain lobes of healthy individuals (Biochain, Newark, CA: R1234062-P, R1234078-P, R1234051-P, R1234066-P) was used as control. In particular, a total of four different pools were used, each containing total RNA from five different healthy donors. RNeasy Mini Kit (Qiagen) was used to extract total RNA from cell lines. Nanodrop spectrophotometer was used to quantify extracted RNA. Using the High-Capacity cDNA Reverse Transcription Kit (Thermo Fisher Scientific), 1 µg of total RNA was reverse transcribed into cDNA following the manufacturer’s protocol. Real-time PCR was performed on 10 ng of cDNA per reaction, with QuantStudio1 Real-time PCR system (Thermo Fisher Scientifc) using TaqMan Universal Master Mix II (Thermo Fisher Scientific) and TaqMan probes. GAPDH and 18S were used as housekeeping genes for cell lines and tissue samples, respectively. Data were presented as fold changes relative to the expression levels of control samples in accordance with the 2^−ΔΔCt^ formula. Validated gene probes used are: 18S Hs99999901_s1, GAPDH Hs99999905_m1, PLCβ1 Hs01001930_m1 (Thermo Fisher Scientific).

### Protein extraction and Western blot

Cells were lysed with T-PER lysis buffer supplemented with Halt protease and phosphatase inhibitor cocktails (Thermo Fisher Scientifc). Lysed cells were sonicated for 15 s using 40–50% of power. Whole cell lysates were quantified with the Pierce™ Coomassie Plus (Bradford) Assay Reagent (Thermo Fisher Scientific) and 30 µg of total proteins were separated on bolt 4–12% polyacrylamide-0.1% commercial SDS gels (Thermo Fisher Scientific) and transferred onto nitrocellulose membrane. Membranes were washed with PBS-0.1% Tween-20 (PBST) and non-specific binding sites were blocked with blocking buffer (5% w/v non-fat dry milk in PBST) for 1 h at room temperature. Lastly, membranes were incubated with primary antibodies overnight at 4 °C. The antibodies used were diluted in bovine serum albumin (BSA) (Sigma-Aldrich) or milk following manufacturer’s protocols. Membranes were washed again with PBST, then incubated with peroxidase conjugated secondary antibodies (Thermo Fisher Scientifc) diluted 1:1500 (for anti-Mouse) and 1:10’000 (for anti-Rabbit) in PBST for 1 h at room temperature. Westar Antares and Westar Supernova (Cyanagen, Bologna, Italy) were used as chemiluminescence reagents to detect immunoreactive bands. Images were captured with the iBright Imaging System (Thermo Fisher Scientific) and samples were analysed by total protein normalization through the iBright analysis software. The following antibodies were used in Western blotting: anti-PLCβ1 (PA5-78430, Thermo Fisher Scientific), anti-Slug (sc-166476, SantaCruz Biotechnology, Dallas, Texas, US), anti-N-Cadherin (33–3900, Thermo Fisher Scientific), anti-MMP-2 (CST40994, Cell Signaling Technology, Danvers, MA, US), anti-MMP-9 (MA5-32705, Thermo Fisher Scientific), anti- β-Catenin (CST9587, Cell Signaling Technology), anti-Non-phospho (Active) β-Catenin (CST8814, Cell Signaling Technology), anti-C-myc/N-myc (CST13987, Cell Signaling Technology), anti-PPARγ (CST2443, Cell Signaling Technology), anti-phospho-Stat3 Ser727 (CST9134, Cell Signaling Technology), anti-phospho-Stat3 Tyr705 (CST9131, Cell Signaling Technology), anti-p44/42 MAPK (Erk1/2) (CST4695, Cell Signaling Technology), anti-Phospho-p44/42 MAPK (Erk1/2) (CST4376, Cell Signaling Technology).

### Immunocytochemistry

Cells were fixed in 2% paraformaldehyde at room temperature (RT) for 20 min. After blocking and permeabilizing with 1% BSA and 0,3% Tryton X-100 for 1 h at RT, cells were incubated with primary antibody overnight at 4 °C. Dilutions of primary antibodies were in accordance with the manufacturer’s instructions. Cells were then incubated in the dark at RT for 1 h with corresponding secondary antibodies conjugated to Alexa Fluor plus 555 or Alexa Fluor 488 (Thermo Fisher Scientific). Lastly, nuclei were stained with Hoechst 33,342 (Thermo Fisher Scientifc) and the coverslips were mounted on the slides with Fluoromount-G (Thermo Fisher Scientific). Slides were then examined under a Zeiss AxioImager Z1 fluorescent microscope (Carl Zeiss International, Germany). Different fields were examined at 40× and 63× magnification. The following antibodies were used: anti-PLCβ1 (Thermo Fisher Scientific) diluted 1:100, anti-Ki67 (Cell Signaling Technology) diluted 1:400, anti-Non-phospho (Active) β-Catenin (Cell Signaling Technology) diluted 1:600.

### Transwell migration and invasion assays

All the cells were trypsinized and suspended in serum-free medium. 100 µl of the cell suspensions containing 3 × 10^4^ cells/ml (for migration assay) or 6 × 10^4^ cells/ml (for invasion assay), were seeded into the upper chamber of a 24-well transwell (8 µm pore size) (Sarstedt, Nümbrecht, Germany). For the invasion assay, a coating with Geltrex (Thermo Fisher Scientific) was carried out 2 h before the seeding and the plates with coated transwells were left 1 h at 37 °C and 1 h at room temperature before the seeding. For both migration and invasion assays, after the seeding, transwells were inserted into a 24-well plate containing 600 µl of medium supplemented with 10% FBS and incubated at 37 °C in a humidified atmosphere for 18 h to allow the cells to migrate/invade. The next day, non-migrating and non-invading cells on the upper side of the filter were removed with cotton swabs. Migrating and invading cells on the lower side of the filter were fixed for 20 min using 70% EtOH and stained for 15 min with 0.2% crystal violet. The number of migrating and invading cells was manually counted in 4 random and non-repeated fields under an optical microscope (magnification 20×). The average cell numbers of each group were then calculated. Each assay was performed in triplicate.

### Wound healing assay

Cells were plated in 6-well plates and the day after reaching 100% confluence a longitudinal scratch was made in the monolayer using a 100 µl sterile micropipette tips. Two independent areas of each lesion were photographed at 0 h and 24 h (for U87-MG) or 48 h (for U-251 MG) using a phase contrast microscope (Motic AE21, Seneco Srl, Milano, Italy) with a digital camera (Visicam 3.0) at ×10 magnification. The gap area was quantified with ImageJ software (National Institutes of Health, Bethesda, MD) and the wound healing effect was calculated as (1-Ax/A0) %, where A0 and Ax represented the empty scratch area at 0 h and 24/48 h, respectively.

### Statistical analysis

Statistical analysis was carried out using Graph Pad Prism 5.0 software (San Diego, CA, US) by applying the unpaired *t* test for patient’s RNA expression analysis and one-way ANOVA for the other analysis. The differences were considered significant with *p < 0.05, **p < 0.01 and ***p < 0.001.

## Results

### Histopathological and molecular characterization of glioblastoma samples

All 50 samples recruited for this study with a diagnosis of glioblastoma were characterized following the WHO 2016 and cIMPACT-NOW guidelines[41] (Supplementary Table 1 for details). Among 50 glioblastoma samples, only 6 were detected mutant for isocitrate dehydrogenase 1 (*IDH1*), *p.R132H* and none for isocitrate dehydrogenase 2 (*IDH2*) and histone *H3-3A. *These IDH1-mutated samples will be classified as Adult-type diffuse astrocytoma, IDH mutant, grade 4 considering the recent tumor classification update[46]. The Telomerase Reverse Transcriptase (*TERT*) promoter was found to be mutated in 32 out of 50 cases (32/50) (29 for g.1,295,113 G > A and 3 for g.1,295,135 G > A). O-6-Methylguanine-DNA Methyltransferase (MGMT) was detected hypermethylated in 23 out of 50 cases (23/50).

### Glioblastoma is characterized by reduced PLCβ1 gene expression compared to both low-grade gliomas and healthy patients.

**Fig. 1:**
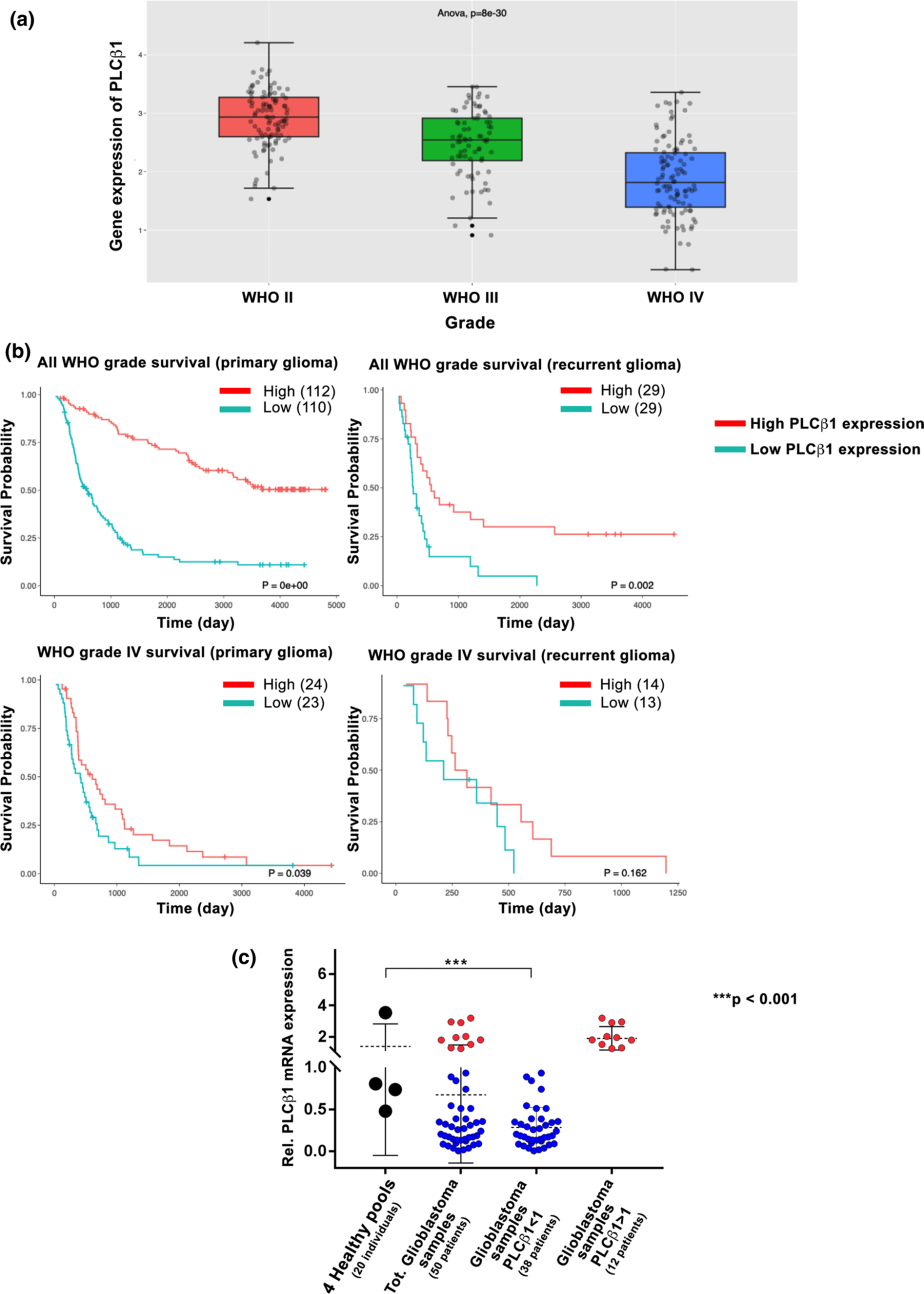
PLCβ1 expression inversely correlates with the pathological grade of gliomas. **a** distribution of PLCβ1 expression in gliomas according to WHO grade status in the CGGA database. WHO II, *n = *103; WHO III, *n = *79; WHO IV, *n = *139. The WHO grading of gliomas inversely correlated with mRNA levels of PLCβ1. **b** Kaplan–Meier survival curves of PLCβ1 high or low expression groups in different glioma patients from the CGGA dataset. Patients were divided according to the median level of PLCβ1 mRNA expression. **c** PLCβ1 mRNA expression in 50 glioblastoma samples and 4 healthy pools of 5 donors each (20 healthy samples in total). Scatter plots display the distribution of PLCβ1 gene expression in glioblastoma samples compared to healthy samples. Glioblastoma patients were stratified based on their PLCβ1 gene expression: patients with higher PLCβ1 expression compared to controls’ mean PLCβ1 expression are represented in red (12 patients), while patients with lower PLCβ1 expression are shown in blue (38 patients). 18S was used as housekeeping gene and the values are presented as mean ± SD. Asterisks indicate statistically significant differences between the groups, with ***p < 0.001

To perform PLCβ1 gene expression study, we first examined the CGGA online public database containing PLCβ1 RNA-seq and survival data of patients with different glioma grades (from II to IV). What emerged from the first analysis was the inverse correlation between the expression of PLCβ1 and the pathological grade of gliomas (Fig. 1a). Indeed, WHO IV gliomas showed a lower gene expression of PLCβ1 compared to WHO II and III gliomas, confirming data already present in literature. In addition, the results of the survival analysis carried out with the CGGA database indicated that patients with low- or high- grade glioma, characterized by a low expression of PLCβ1 have a shorter survival time in both primary and recurrent gliomas compared to patients with high-PLCβ1 expression (Fig. 1b).

Subsequently, we analyzed PLCβ1 gene expression in 50 tissues from fresh-frozen retrospective glioblastoma patients of the last 10 years, compared to 4 healthy samples pools, each one containing total RNA derived from 5 different healthy donors, used as controls (20 patients in total). As shown in the graph (Fig. 1c), it was highlighted an overall lower expression of PLCβ1 in glioblastoma samples compared to the healthy ones. To further investigate this result, we stratified patients on their PLCβ1 expression, obtaining 38 patients out of 50 (38/50) with lower PLCβ1 expression compared to the PLCβ1 mean expression of the healthy pools (PLCβ1 < 1) and 12 patients out of 50 (12/50) with higher PLCβ1 expression (PLCβ1 > 1) compared to controls’ mean expression. After patients’ stratification, the reduction in PLCβ1 expression resulted to be statistically significant in 38 glioblastoma patients compared to healthy controls. On the other hand, the higher PLCβ1 expression seen in the other 12 glioblastoma patients was not statistically relevant. Based on all these results, we concluded that glioblastoma was characterized by an overall reduced PLCβ1 expression compared to healthy samples and low-grade gliomas.

### PLCβ1 silencing leads to an increase in the expression of mesenchymal markers and matrix metalloproteinases

**Fig. 2:**
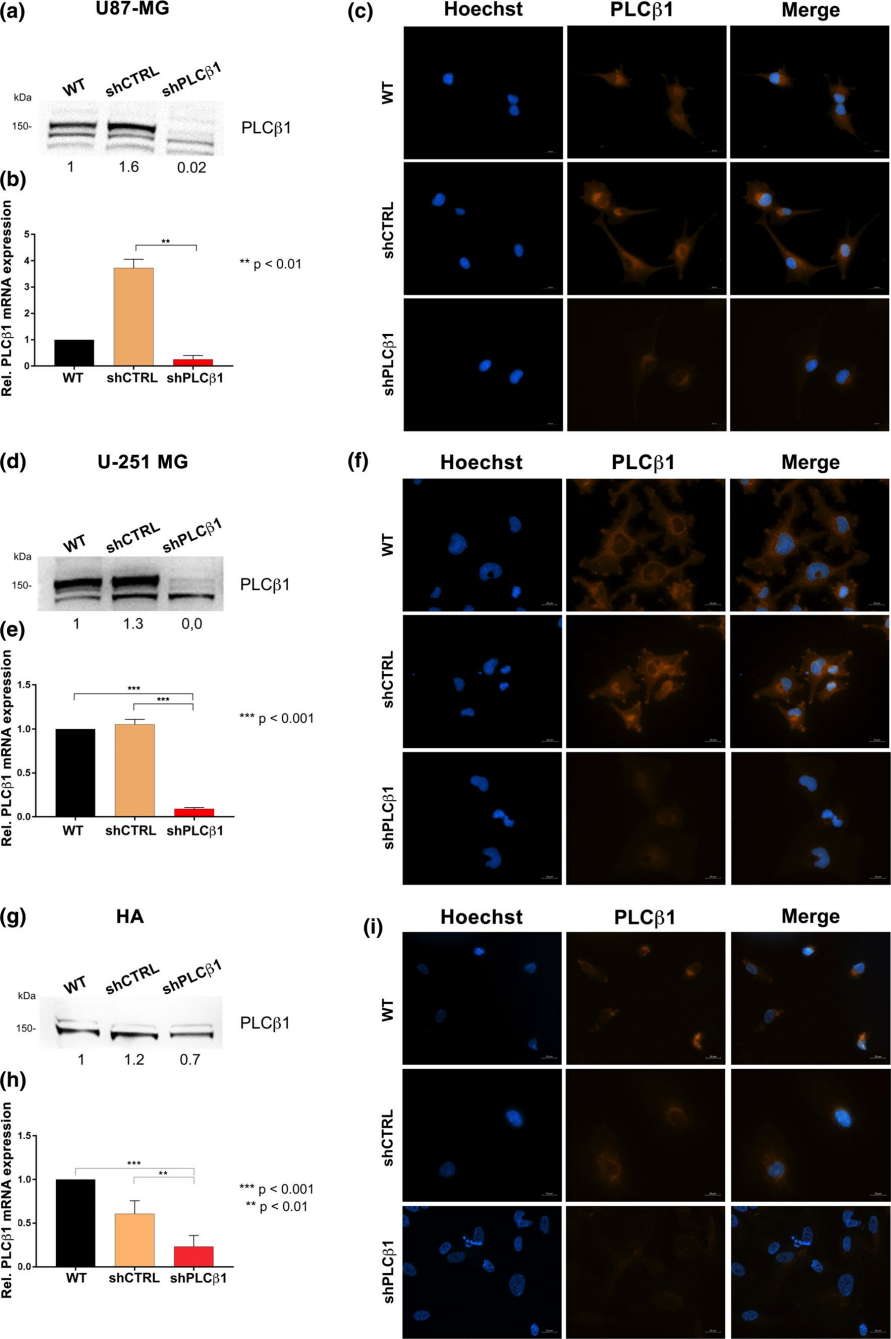
PLCβ1 silencing on U87-MG, U-251 MG cell lines and HA primary astrocytes. Following transduction and antibiotic selection, PLCβ1 mRNA expression, protein levels and localization were evaluated in U87-MG, U-251 MG cells and HA primary astrocytes. PLCβ1-silenced cells (shPLCβ1) were compared to wild type (WT) and mock-transduced cells (shCTRL). U87-MG and U-251 MG cells were tested after one month of antibiotic selection, while HA primary astrocytes were analyzed after 48 h of puromycin selection, i.e. 96 h after transduction overall. **a**, **d**, **g** Western blot analysis of PLCβ1 expression in U87-MG (**a**), U-251 MG (**d**) and HA primary astrocytes (**g**). Densitometric analysis was performed with total protein normalization through the iBright analysis software. Western blot results are representative of three independent experiments. **b**, **e** and **h** PLCβ1 mRNA expression in U87-MG (**b**), U-251 MG (**e**) and HA primary astrocytes (**h**). GAPDH was used as housekeeping gene and all the analysis derived from three independent experiments, with **p < 0.01, ***p < 0.001. **c**, **f** and **i** immunofluorescence staining of PLCβ1 (red) in U87-MG (**c**), U-251 MG (**f**) and HA primary astrocytes (**i**) (magnification ×40, bar: 20 μm). Nuclei were stained with Hoechst 33,342 (blue). Results are representative of at least five different fields

To investigate the relation between PLCβ1 and glioblastoma, we silenced PLCβ1 in two different glioblastoma cell line models. First, we transduced U87-MG and U-251 MG cell lines and we created stable clones after a month of selection with puromycin. After this period, silenced cells (shPLCβ1 cells) were tested for PLCβ1 protein (Fig. 2a, d and Supplementary Fig. 1a, b) and gene expression (Fig. 2b, e) and compared to wild type cells (WT) and to cells transduced with empty vectors coding only for GFP (shCTRL). In addition, PLCβ1 expression and localization were evaluated by immunocytochemistry (ICC) (Fig. 2c, f). Next, to better deepen our study, we introduced a new cell model in our in vitro experiment, based on human primary astrocytes (HA). HA were analyzed for PLCβ1 protein (Fig. 2g and Supplementary Fig. 1c), gene expression (Fig. 2h), and localization (Fig. 2i), 24 h after puromycin treatment and 96 h after the transduction, revealing a slightly lower efficiency than cell lines.

**Fig. 3:**
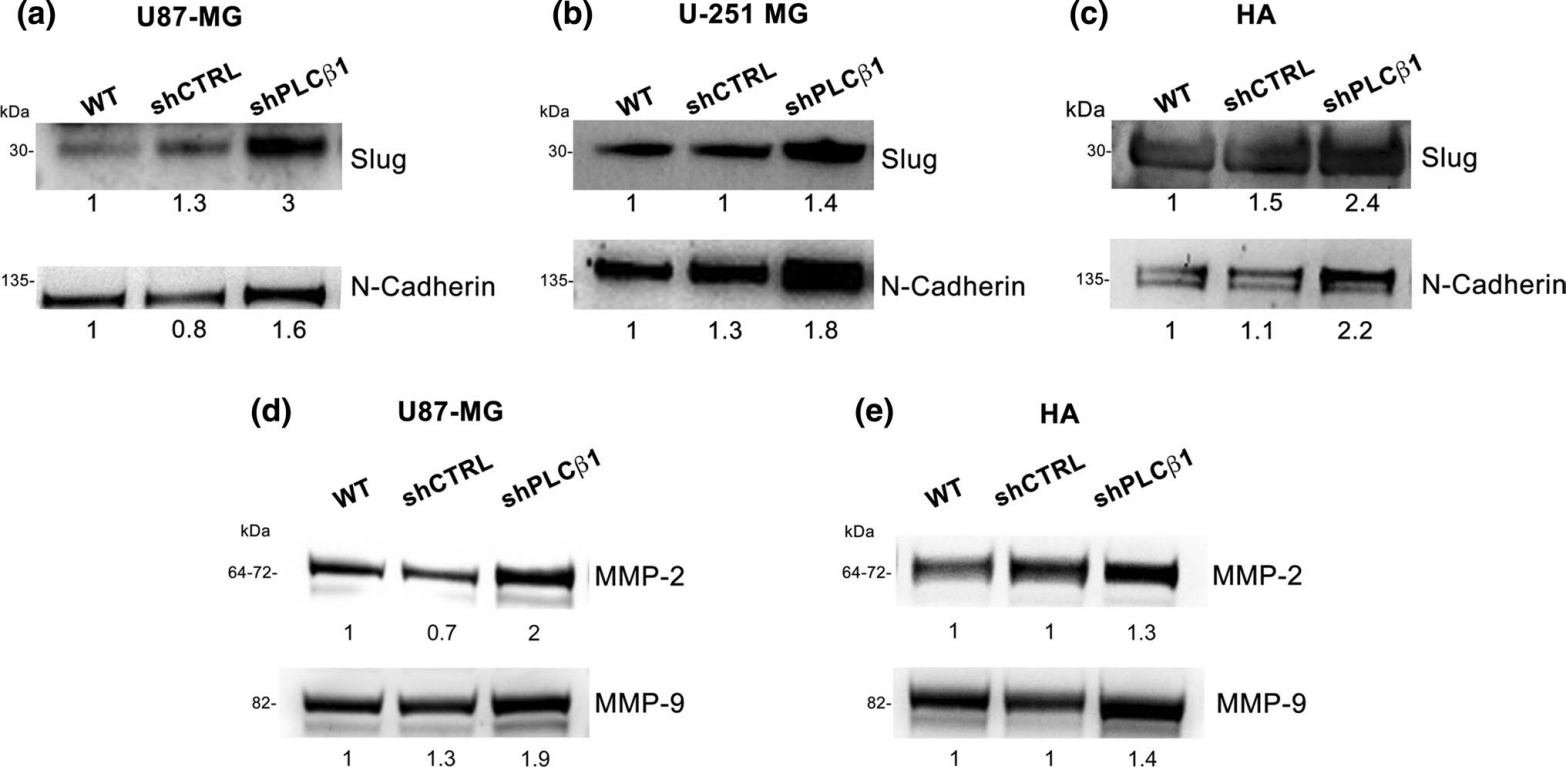
PLCβ1 silencing leads to increased mesenchymal markers and matrix metalloproteinases expression. **a**, **b** and **c** Western blot analysis of the expression of mesenchymal markers Slug and N-Cadherin after PLCβ1 silencing on U87-MG (**a**), U-251 MG (**b**) and HA primary astrocytes (**c**). **d** and **e** Western blot analysis of matrix metalloproteinases MMP-2 and MMP-9 expression on U87-MG (**d**) and HA primary astrocytes (**e**). PLCβ1-silenced cells (shPLCβ1) were compared to wild type (WT) and mock-transduced (shCTRL) samples. Densitometric analysis was performed with total protein normalization through the iBright analysis software. Western blot results are representative of three independent experiments

To investigate the effects of reduced PLCβ1 on the main molecular mechanisms that regulate cancer development, we examined the physiological and pathological processes required for cell migration and invasion. We evaluated the expression of the mesenchymal phenotype-associated molecule N-Cadherin and of one of the main epithelial-mesenchymal transition (EMT) regulatory transcription factors Slug. Following stable or transient PLCβ1 silencing, Western blot analysis revealed that both cell lines and primary astrocytes show an increase in the expression of the two markers, revealing that PLCβ1 silencing determines a more undifferentiated state compared to controls. Indeed, the mesenchymal markers were upregulated in all PLCβ1-silenced models (Fig. 3a, b, c and Supplementary Fig. 2a, b, c).

Moreover, following PLCβ1 silencing, we also observed a significant higher protein expression of metalloproteinases 2 (MMP-2) and 9 (MMP-9), which are involved in extracellular matrix (ECM) degradation and in the epithelial-mesenchymal transition, in U87-MG and HA primary astrocytes (Fig. 3d, e and Supplementary Fig. 2d, e). Data were not shown on U-251 MG due to the lack of expression of MMPs, which were not detectable in this cell line. All in all, these data suggested that PLCβ1 silencing led to a more aggressive phenotype.

### Migration, invasion and proliferation abilities are increased in PLCβ1-silenced cells

**Fig. 4:**
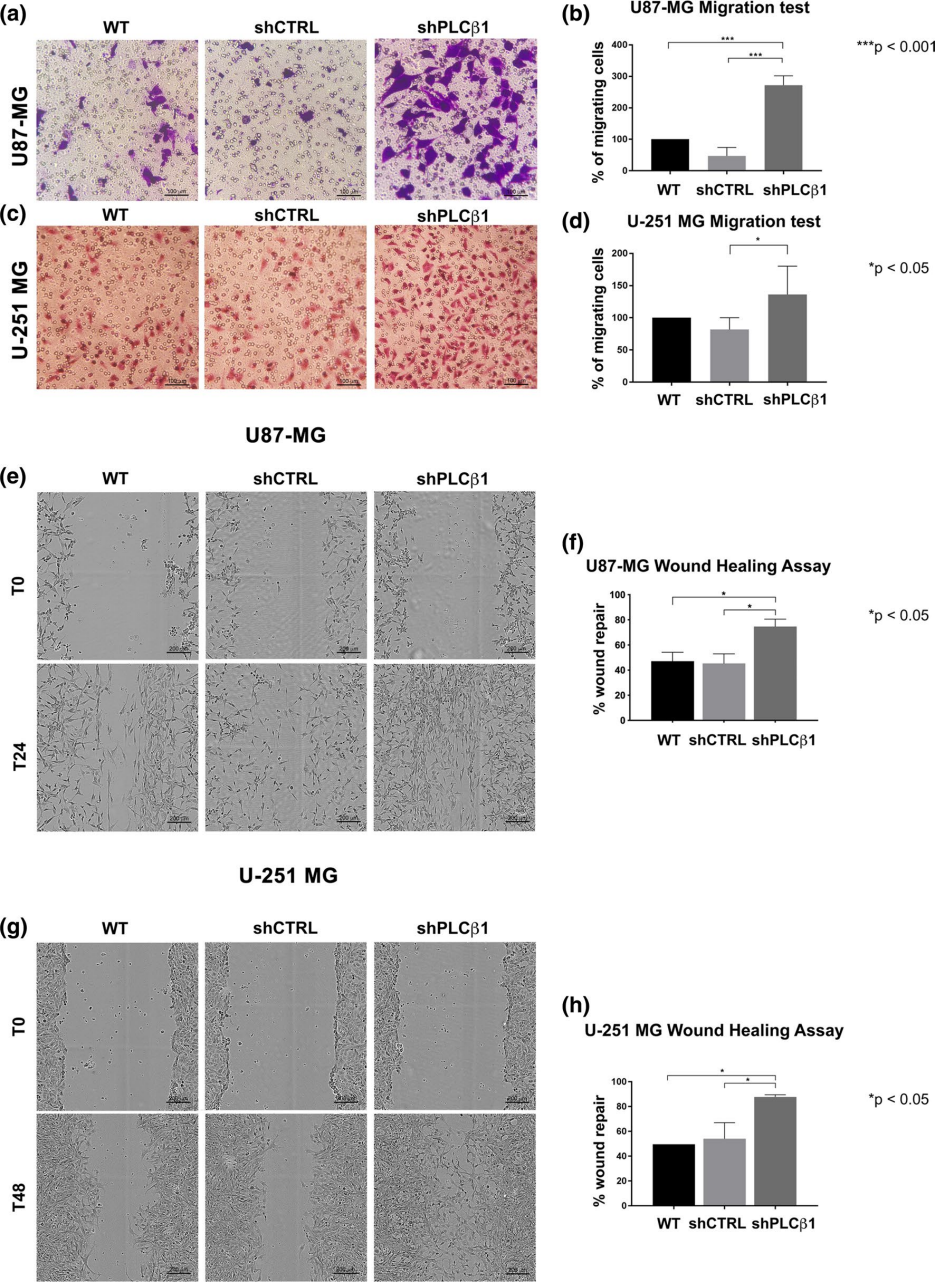
PLCβ1 silencing causes increased cell migration in U87-MG and U-251 MG cell lines. **a** and **c** representative images of transwell migration assays in U87-MG (**a**) and U-251 MG (**c**) cell lines. PLCβ1-silenced cells (shPLCβ1) were compared to wild type (WT) and mock-transduced (shCTRL) cells. Magnification ×20 (bar: 100 μm). **b** and **d** graphical representations of transwell migration assays of PLCβ1-silenced cells (shPLCβ1) compared to wild type (WT) and mock-transduced (shCTRL) cells in U87-MG (**b**) and U-251 MG (**d**) cell lines. Columns show the mean ± SD of three independent experiments with *p < 0.05, ***p < 0.001. **e** and **g** wound healing assays of U87-MG (**e**) and U-251 MG (**g**) cell lines. PLCβ1-silenced cells (shPLCβ1) were compared to wild type (WT) and mock-transduced (shCTRL) cells. Representative pictures were taken at 0 h, 24 h (for U87-MG) and 0 h, 48 h (for U-251 MG) after scratching. Magnification ×10 (bar: 200 μm). **f** and **h** graphical representations of wound healing assays of PLCβ1-silenced cells (shPLCβ1) compared to wild type (WT) and mock-transduced (shCTRL) cells in U87-MG (**f**) and U-251 MG (**h**) cell lines. Columns show the mean ± SD of three independent experiments with *p < 0.05

**Fig. 5:**
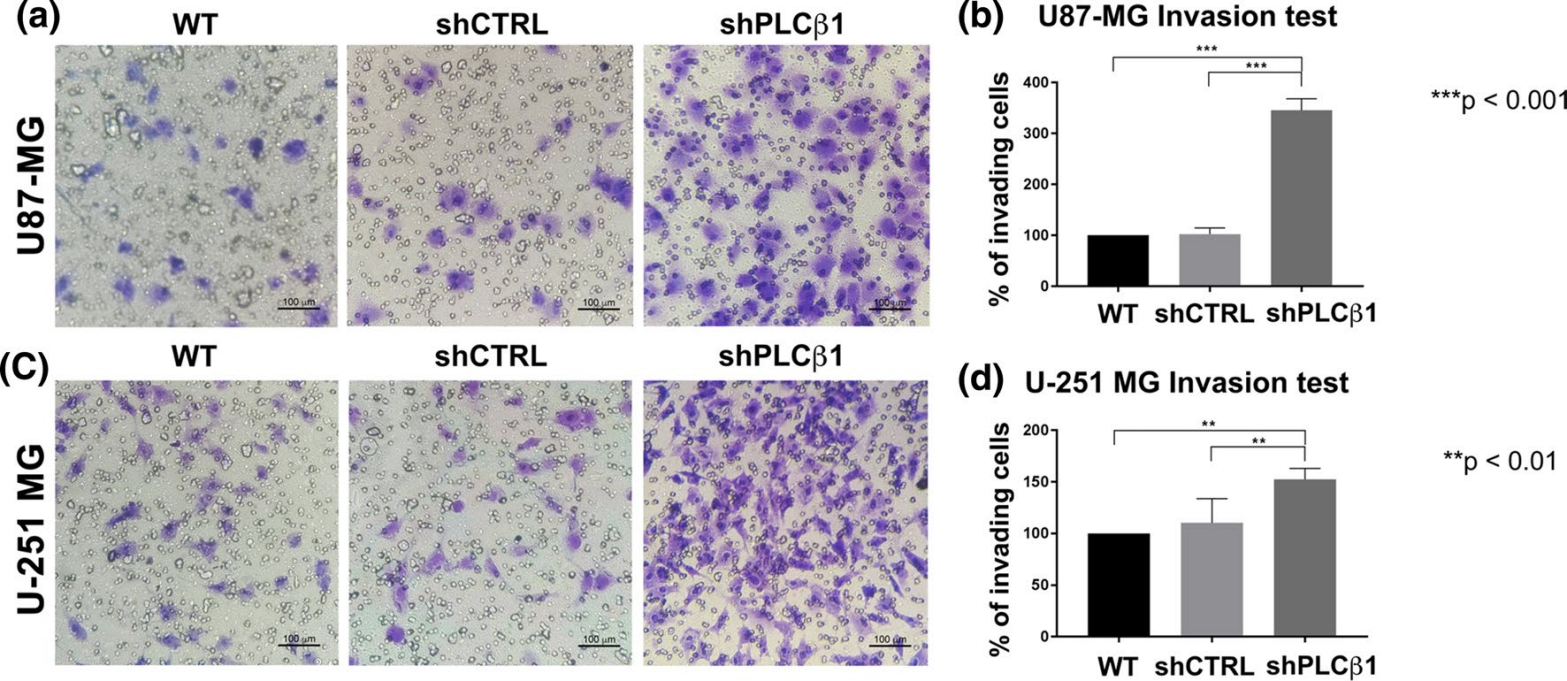
PLCβ1 silencing determines increased cell invasion in U87-MG and U-251 MG cell lines. **a** and **c** representative images of transwell invasion assays with Geltrex coating in U87-MG (**a**) and U-251 MG (**c**) cell lines. PLCβ1-silenced cells (shPLCβ1) were compared to wild type (WT) and mock-transduced (shCTRL) cells. Magnification ×20 (bar: 100 μm). **b** and **d** graphical representations of transwell invasion assays of PLCβ1-silenced cells (shPLCβ1) compared to wild type (WT) and mock-transduced (shCTRL) cells in U87-MG (**b**) and U-251 MG (**d**) cell lines. Columns show the mean ± SD of three independent experiments with **p < 0.01***p < 0.001

**Fig. 6:**
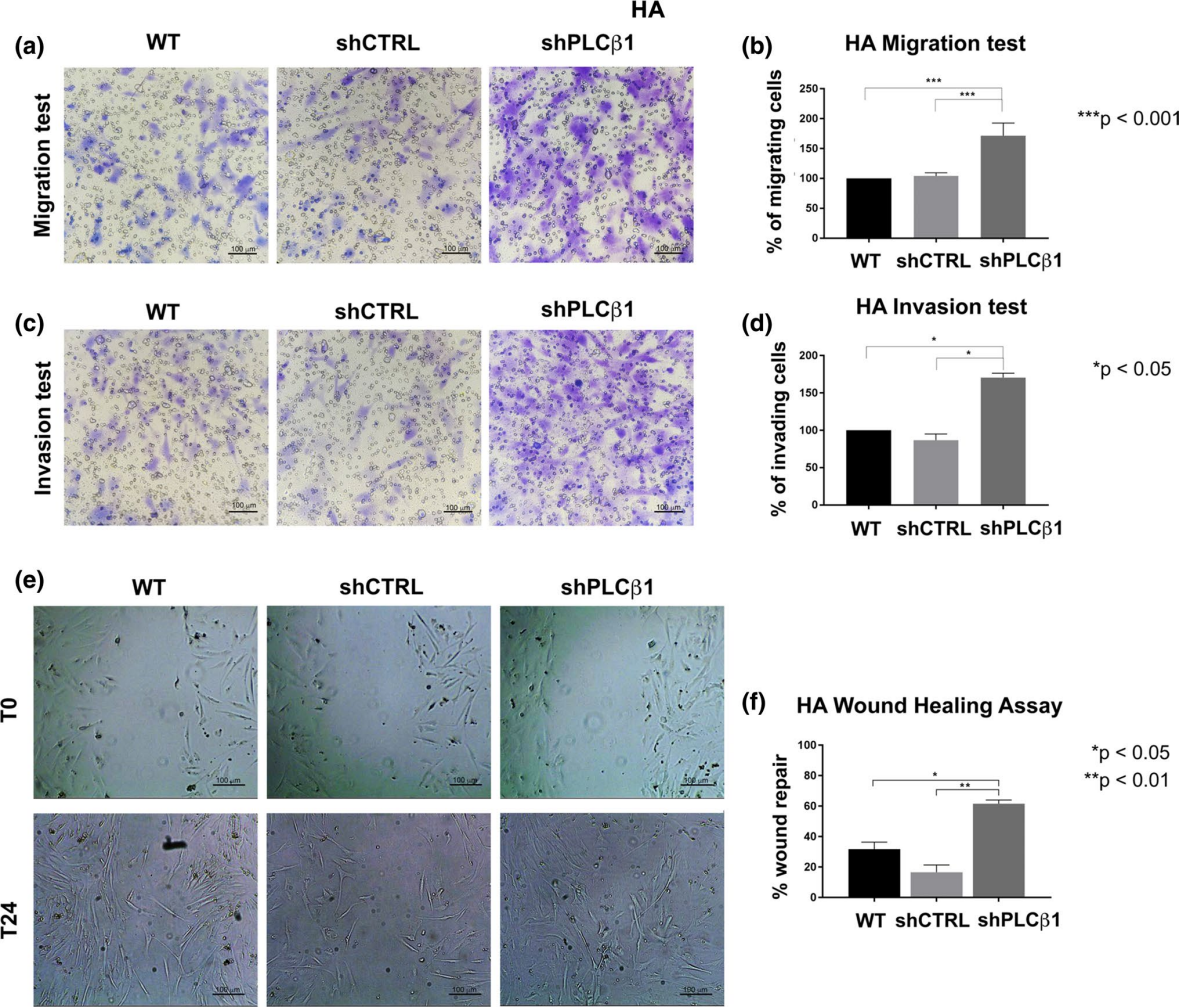
PLCβ1 silencing causes increased cell migration and invasion in HA primary astrocytes.** a** Representative images of transwell migration assay in HA primary astrocytes. PLCβ1-silenced cells (shPLCβ1) were compared to wild type (WT) and mock-transduced (shCTRL) cells. Magnification ×20 (bar: 100 μm). **b** Graphical representation of transwell migration assay of PLCβ1-silenced cells (shPLCβ1) compared to wild type (WT) and mock-transduced (shCTRL) cells. Columns show the mean ± SD of three independent experiments with ***p < 0.001. **c** Representative images of transwell invasion assay with Geltrex coating in HA primary astrocytes. PLCβ1-silenced cells (shPLCβ1) were compared to wild type (WT) and mock-transduced (shCTRL) cells. Magnification ×20 (bar: 100 μm). **d** Graphical representation of transwell invasion assay of PLCβ1-silenced cells (shPLCβ1) compared to wild type (WT) and mock-transduced (shCTRL) cells. Columns show the mean ± SD of three independent experiments with *p < 0.05. **e** Wound healing assay of HA primary astrocytes. PLCβ1-silenced cells (shPLCβ1) were compared to wild type (WT) and mock-transduced (shCTRL) cells. Representative pictures were taken at 0 h and 24 h after scratching. Magnification ×10 (bar: 100 μm). **f** Graphical representation of wound healing assays of PLCβ1-silenced cells (shPLCβ1) compared to wild type (WT) and mock-transduced (shCTRL) cells. Columns show the mean ± SD of three independent experiments with *p < 0.05 and **p < 0.01

Based on the previous data, we next examined the migration and invasion potential of PLCβ1-silenced cells. Following PLCβ1 silencing, both U87-MG and U-251 MG cells exhibited significantly higher migration ability compared to their respective controls (shCTRL) and wild type cells (WT). This result was confirmed by both transwell migration assay (Fig. 4a, b, c, d), and wound healing assay (Fig. 4e, f, g, h). In particular, based on the latter results, PLCβ1-silenced U87-MG and U-251 MG cells were able to almost completely repair the wound after 24 h and 48 h, respectively, while their controls, failed to totally repair the wound in the same time frame. Next, to evaluate the invasion abilities of U87-MG and U-251 MG, we performed the transwell assay with a Geltrex coating. This revealed that both PLCβ1-silenced cell lines had significantly higher invasion ability than the respective controls (Fig. 5a, b, c, d). The same assays were further carried out on HA primary astrocytes. PLCβ1-silenced astrocytes were able to migrate faster (Fig. 6a, b), invade faster (Fig. 6c, d) and almost completely repair the wound (Fig. 6e, f) compared to control and wild type cells. Therefore, an inverse correlation between PLCβ1 expression levels and the cellular migration and invasion abilities was confirmed in all cell models.

**Fig. 7:**
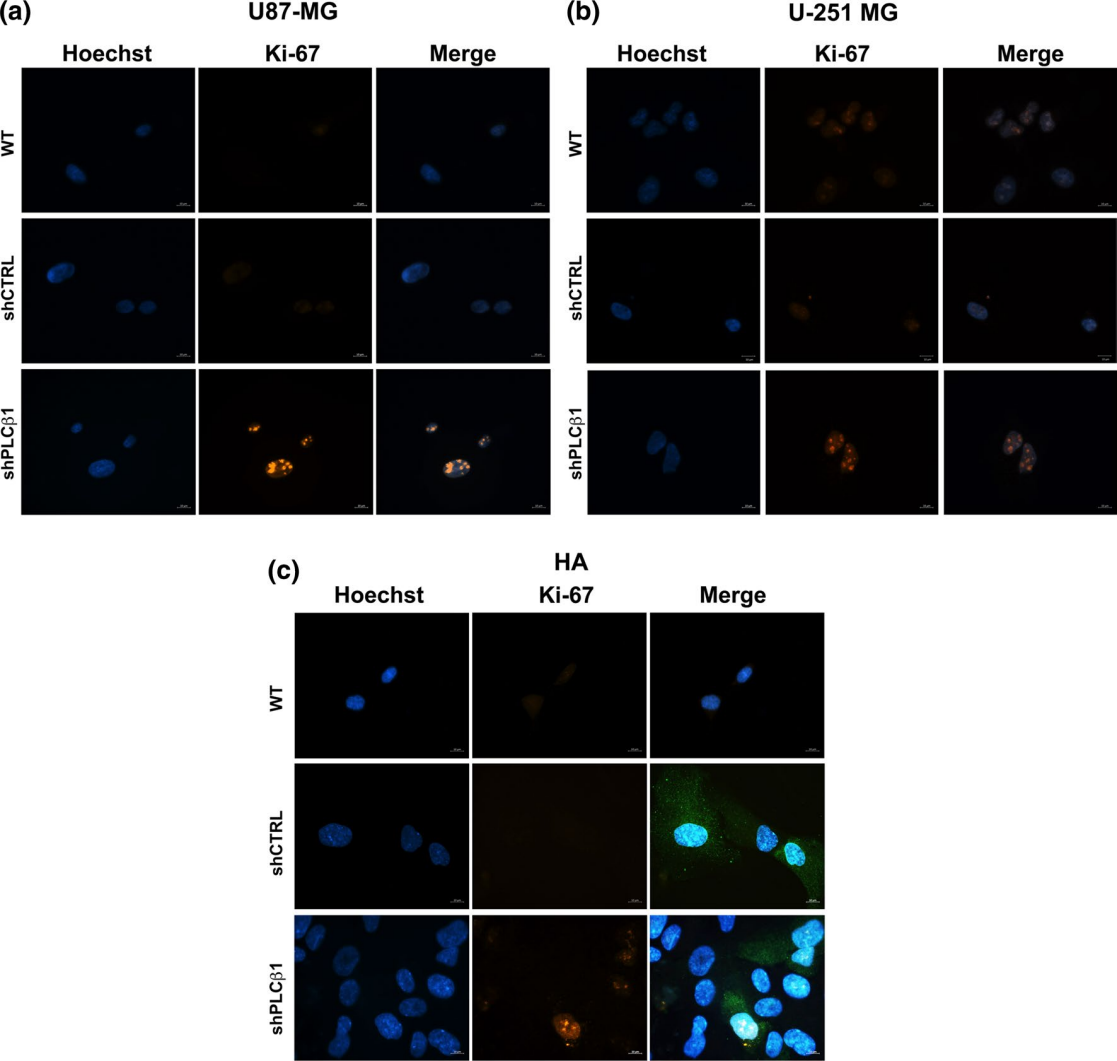
PLCβ1 silencing leads to increased cell proliferation. **a** and** b** Immunofluorescence staining of Ki-67 (red) at ×63 magnification (bar: 10 μm) in U87-MG (**a**) and U-251 MG (**b**). **c** Immunofluorescence staining of Ki-67 (red) and GFP (green) at ×63 magnification (bar: 10 μm) in HA primary astrocytes. PLCβ1-silenced cells (shPLCβ1) were compared to wild type (WT) and mock-transduced (shCTRL) cells. Nuclei were stained using Hoechst 33,342 (blue). Results are representative of at least five different fields

To verify if PLCβ1 silencing is also associated with increased proliferation, we evaluated the expression of the proliferation marker Ki-67 by immunofluorescence. Figure 7 displays the expression of Ki-67 in wild type (WT), mock-transduced (shCTRL), and PLCβ1-silenced cells (shPLCβ1). Both U87-MG and U-251 MG showed a higher expression of Ki-67 (in red) in PLCβ1-silenced cells compared to their controls and wild type cells (Fig. 7a, b). Next, we evaluated Ki-67 expression in HA primary astrocytes. Since this model showed a lower transduction efficiency compared to cell lines, we also acquired the GFP signal (in green) to identify transduced cells. Comparing Ki-67 expression in GFP + cells, it was confirmed that shPLCβ1 HA had higher expression of Ki-67 compared to shCTRL and wild type cells (Fig. 7c). Therefore, cell proliferation is markedly affected by PLCβ1 reduced expression both in cell lines and in primary astrocytes.

### PLCβ1 silencing enhances the activation of survival pathways

**Fig. 8:**
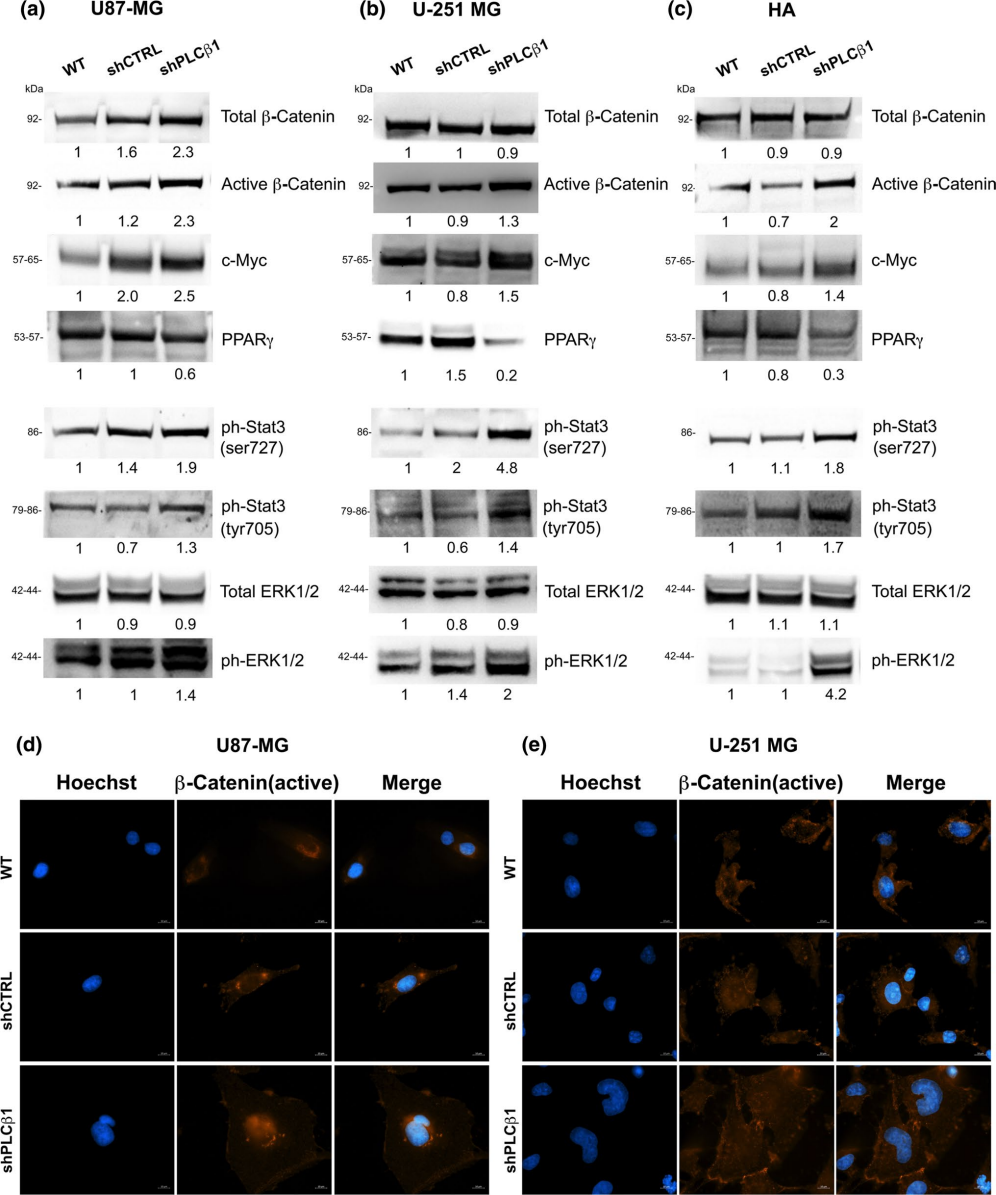
Effects of PLCβ1 silencing on the activation of survival pathways. **a**, **b** and **c** The expression and the phosphorylation of molecules belonging to different survival pathways were evaluated in U87-MG (**a**), U-251 MG (**b**) and HA primary astrocytes (**c**). PLCβ1-silenced cells (shPLCβ1) were compared to wild type (WT) and mock-transduced (shCTRL) cells. Densitometric analysis was performed with total protein normalization through the iBright analysis software. Western blot results are representative of three independent experiments. **d** and **e** Immunofluorescence staining of active β-catenin (red) at ×63 magnification (bar: 10 μm) in U87-MG (**d**) and U-251 MG (**e**). PLCβ1-silenced cells (shPLCβ1) were compared to wild type (WT) and mock-transduced (shCTRL) cells. Nuclei were stained using Hoechst 33,342 (blue). Results are representative of at least five different fields

Next, we investigated the signaling molecules involved in different survival pathways, such as β-catenin, Stat3 and ERK1/2 pathways, to determine if these signaling cascades were influenced by PLCβ1 expression. Figure 8a, b, together with supplementary Fig. 3a, b, show that in both U87-MG and U-251 MG cells, PLCβ1 silencing led to an increase in the activation of survival pathways. In both cell lines, it was possible to observe an increment in the expression of the active form of β-Catenin (non-phosphorylated form on residues Serine 33, Serine 37 and Threonine 41) and, in U87-MG, an increase of the total one, following PLCβ1 silencing. We also evaluated the protein expression of the proto-oncogene c-Myc, as a well-known β-catenin target to determine if the amount of active β-catenin affected the expression of its targets. Indeed, PLCβ1-silenced cells showed a marked increase of c-Myc expression in both cell lines compared to control samples (WT and shCTRL). Moreover, an immunocytochemical study of the active form of β-catenin in the cell lines revealed a nuclear presence of active β-catenin in PLCβ1-silenced cells (Fig. 8d,e), particularly evident in U87-MG. Indeed, while wild type and control cells showed a comparable diffuse cytoplasmic localization of active β-catenin, shPLCβ1 cells revealed a concomitant nuclear presence of it. This confirmed that the active form of β-catenin translocated into the nucleus to act as a transcriptional activator in U87-MG and U-251 MG cell lines, following PLCβ1 silencing. Next, we investigated the effect of PLCβ1 silencing on the expression of the nuclear receptor Peroxisome Proliferator-Activated Receptor γ (PPARγ), which is known to act in opposition to β-catenin in several cellular models. Figure 8 shows that PLCβ1-silenced cells display a decrease of PPARγ. This downregulation is more marked in U-251 MG compared to U87-MG. In addition, it was observed that the activation of Stat3 pathway was increased in PLCβ1-silenced cells compared to control and wild type cells. Both U87-MG and U-251 MG cells displayed high levels of phosphorylated Stat3 (both on Serine 727 and Tyrosine 705) compared to control samples. Finally, also the phosphorylation levels of p44/42 MAPK (ERK1/2) were affected by PLCβ1 silencing. Indeed, shPLCβ1 cells showed increased phosphorylation of ERK1/2.

All these data were later strengthened and confirmed also in HA primary astrocytes (Fig. 8c and Supplementary Fig. 3c). Indeed, as well as in U87-MG and U-251 MG, also in primary HA model, a marked increase of the active form of β-catenin was observed, together with the increase of its target c-Myc, as a consequence of PLCβ1 silencing. Furthermore, a decrease in the expression of PPARγ, was also observed in PLCβ1-silenced cells. Moreover, in support of these data, it was also found an increased expression of the phosphorylated forms of Stat3 and of ERK1/2.

## Discussion

Glioblastoma is the most common malignant brain tumor in adults. Despite all the extensive studies on glioblastoma worldwide, current treatment therapies are still not successful, highly invasive and cannot cure definitively the tumor. Indeed, this kind of tumors develop resistance to treatments and recur quickly, due to the great tumor complexity and heterogeneity [47]. For this reason, the management of glioblastoma represents today a great challenge and the understanding of the molecular mechanisms related to tumor transformation could help to find a successful targeted therapeutic strategy. Phosphoinositide (PI) metabolic enzymes, especially phospholipases C (PLCs) have been reported to be involved in many tumor mechanisms, such as cell proliferation, differentiation, migration and cell cycle [48] and in many physio-pathological brain processes [15, 49]. It has been also documented that some PLCs are involved in several brain disorders including epilepsy, movement and behavior disorders, neurodegenerative diseases and also high-grade gliomas [15]. Indeed, a previous in silico study reported that PLCβ1, one of the most represented PLCs in the brain, is a potential prognostic factor and accordingly, a candidate signature gene for specific subtypes of glioblastoma and that PLCβ1 expression was inversely correlated with glioma pathological grades [26]. Moreover, in the same study, it was demonstrated that glioma patients with intermediate PLCβ1 expression survived significantly longer than PLCβ1 downregulated group [26]. Another enzyme that has been recently identified as a key element for this tumor aggression mechanisms is the PLCγ1 [25]. Through a translational study, which combined data from patients’ samples with data on U87-MG engineered cell line, it was demonstrated a strong correlation between PLCγ1 expression level and the acquisition of a more aggressive cellular phenotype [25].

Although PLCβ1 and PLCγ1 appear to have an opposite trend in glioblastoma, these two enzyme isoforms do not seem to have a compensatory trend between each other (Supplementary Fig. 4), highlighting the importance of investigating deeply the pathological or protective role of phospholipases and their intermediaries in glioblastoma, making them potential targets in the search for new therapeutic approaches.

Indeed, our study focused on searching key molecules in the processes of tumor pathological development that can later be used as diagnostic or prognostic factors in high-grade gliomas, which are characterized by a great biological heterogeneity. Therefore, we confirmed and enriched the previous in silico data, using a different analysis platform: the Chinese Glioma Genome Atlas (CGGA) RNA sequencing (RNA-seq) dataset (mRNAseq_325) with 325 glioma samples. Interestingly, this study confirmed that PLCβ1 gene expression was significantly reduced in all WHO IV gliomas (glioblastoma), not only in specific subtypes, compared to WHO II and WHO III gliomas, suggesting a strongly pathological role of PLCβ1 low expression. Furthermore, Kaplan–Meier survival curves from CGGA dataset, demonstrated that patients with low- or high- grade gliomas, characterized by a low expression of PLCβ1, have a shorter survival time in both primary and recurrent gliomas compared to patients with high-PLCβ1 expression. These data confirmed that PLCβ1 expression levels are inversely correlated with glioma pathological grades and that PLCβ1 low levels may be related to a worse prognosis for patients. These in silico data were strengthened by the retrospective analysis of PLCβ1 mRNA expression in 50 fresh-frozen glioblastoma tissue samples in comparison with 20 healthy individuals divided into 4 different pools. This analysis confirmed that PLCβ1 expression is decreased in glioblastoma samples not only compared to low-grade tumors, but also compared to healthy individuals.

To verify the pathological effects of PLCβ1 downregulation, two different *in* *vitro *models were created by PLCβ1 stable knock-down on U87-MG and U-251 MG (glioblastoma immortalized cell lines). Moreover, a transient silencing was also performed on human primary astrocytes (HA), to mimic and best represent the tumor heterogeneity, working on both engineered tumor cell lines and normal astrocytes.

Since tumor invasion, migration and metastasis are the main causes of resistance to current therapies, we focused the first analysis on these tumor mechanisms. It is known that during metastasis formation, glioblastoma cells are characterized by molecular and morphological changes shifting the tumor towards a more undifferentiated state, acquiring mesenchymal characters, including the remodeling and degradation of the ECM [50]. For this reason, we focused our study on the expression analysis of some of the main mesenchymal markers and elements of ECM degradation. Interestingly, in both tumor and normal astrocyte models silenced for PLCβ1, it was observed an increased expression of Slug, an essential transcriptional factor that is involved in the regulation of mesenchymal phenotype, and N-Cadherin, one of the main mesenchymal markers. In addition, since the main driver of ECM degradation is proteolytic digestion by matrix metalloproteinases (MMPs), which expression is strongly linked to the acquisition of a mesenchymal phenotype [50], we evaluated MMP-2 and MMP-9, which expression increased following PLCβ1 silencing in our models. These data were further enhanced by the increment of migration and invasion abilities of our PLCβ1-silenced cells, demonstrating that PLCβ1 silencing in glioblastoma seems to promote a shift towards a more aggressive cellular phenotype. In addition, also cell proliferation is affected by PLCβ1 silencing, as demonstrated by the increased expression of the nuclear marker Ki-67 in all the silenced cell models compared to the controls. Indeed, Ki-67 is widely used as a stable marker of cell proliferation in many types of human tumors, including malignant gliomas [51].

It is well known the role of β-Catenin, a component of the cell–cell adhesion complex, in the regulation of proliferation and migration in different cell types and cancers [37], and interestingly, the active form of this protein is overexpressed in all our PLCβ1-silenced models. It is evidenced that, following PLCβ1 silencing in our models, the translocation of active β-catenin into the cell nucleus is favored, as can be seen from the immunofluorescence (IF) analysis. As a result, this event leads β-catenin to recruit, in the nucleus, transcriptional factors and to regulate the activation of different target genes linked to proliferation and invasion, such as: c-Myc and MMPs, both of which increase in our PLCβ1-silenced models. Moreover, we showed that, following the silencing of PLCβ1, there is a consequent increment in ERK1/2 pathway activation, that is a fundamental pro-surviving factor involved in tumor progression and resistance to current therapies [52]. In glioma cells, the epidermal growth factor (EGF)/EGFR signaling through ERK1/2 leads to the phosphorylation of α-catenin, which, together with cadherin, forms a complex linked to the cytoskeleton, promoting β-catenin transactivation and glioma cell invasion [53]. Furthermore, considering the key role of PPARγ in the CNS [54] and the well-known crosstalk between this latter and β-catenin pathway [55], we evaluated the consequences of PLCβ1 silencing on its protein expression. Effectively, our PLCβ1-silenced cells showed a decline in PPARγ protein expression compared to control samples, confirming its opposite behavior compared to β-catenin pathway. It is interesting to note that a correlation between PLCβ1 low expression and the decrease of PPARγ expression has been previously evidenced also in pancreatic β cells, affecting insulin secretion [56].

Substantially, our glioblastoma models show how PLCβ1 silencing pushes towards cell survival. Indeed, following PLCβ1 silencing, it is also shown in our data, an increased activation of Stat3 pathway, which is a well-defined oncogenic transcription factor that plays a key role in tumor resistance and aggressive cancer progression in glioblastoma [57]. The increased activation of this pathway, together with ERK1/2 and β-catenin pathways, reinforce the hypothesis that PLCβ1 downregulation in glioblastoma promotes a more aggressive phenotype.

**Fig. 9:**
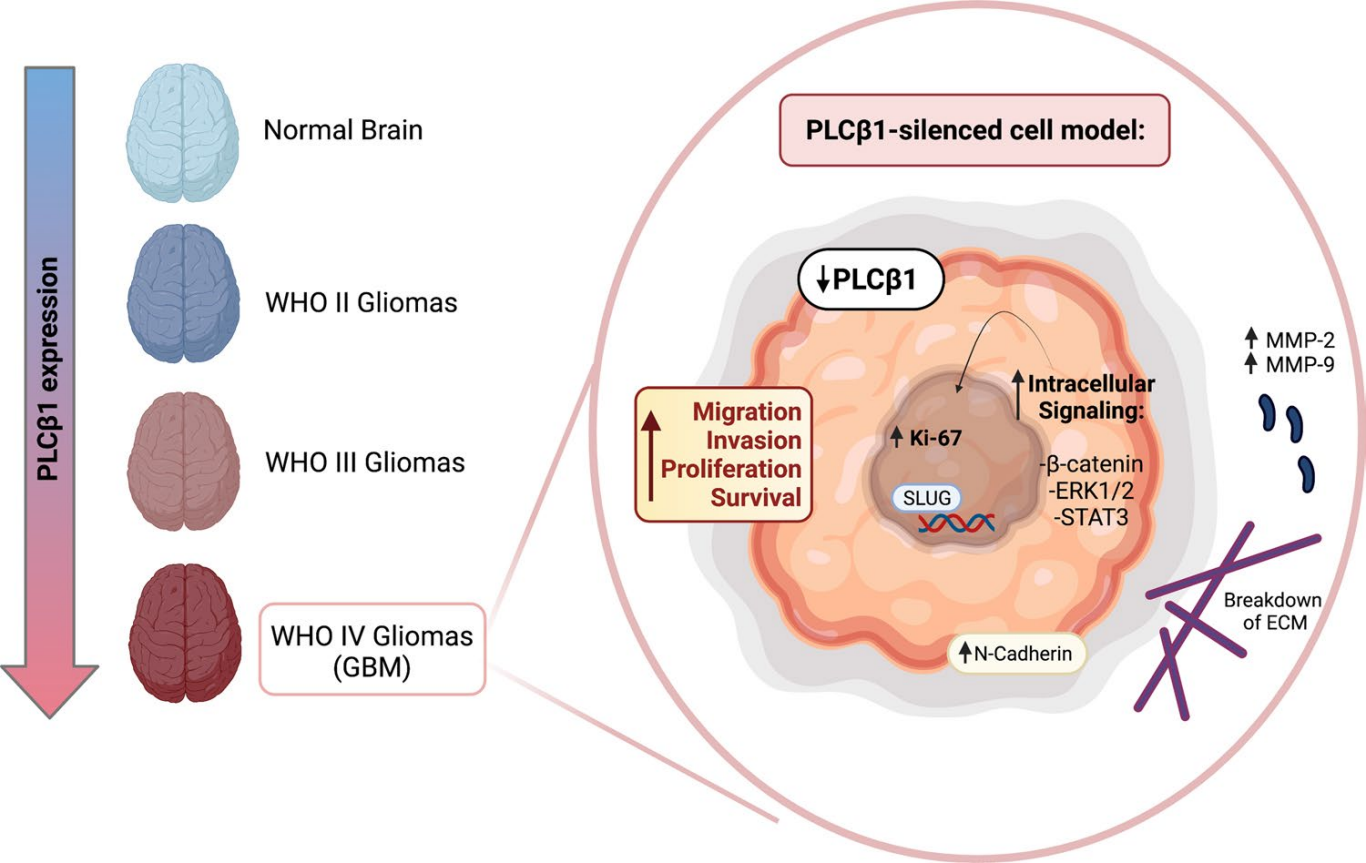
Graphical Abstract which summarizes the possible role of PLCβ1 in Glioblastoma. Image modified from the original article of Lu et al. [26] that shows an inverse correlation between PLCβ1 expression and the pathological grade of gliomas. After confirming this trend, experimental models based on engineered cell lines and primary astrocytes with silenced PLCβ1, were created. PLCβ1 downregulation determines different relevant physio-pathological alterations, leading the cells to acquire a greater ability of migration and invasion, with the relative increment of the expression of some mesenchymal transcription factors and markers, such as Slug and N-Cadherin, and the metalloproteinases MMP-2 and MMP-9. In addition, also cell proliferation, through the increased expression of Ki-67, and the main survival signaling pathways, such as β-catenin, ERK1/2 and Stat3 pathways, are affected by PLCβ1 silencing. All in all, these data suggest a potential role of PLCβ1 in maintaining a normal or less aggressive phenotype of glioma

All in all, in silico data from database, clinical data collected on glioblastoma fresh-frozen samples, together with cellular and molecular data on engineered immortalized and primary cell lines, suggest a potential role of PLCβ1 in maintaining a normal or less aggressive phenotype of glioma (Fig. 9). However, the mechanisms by which PLCβ1 is downregulated in high-grade tumors are not clear yet. Further studies to detect epigenetic anomalies associated with glioblastoma are needed. This step could result essential in the detection of responsible genes that could respond to hypomethylating therapies. This work suggests that PLCβ1 downregulation and the consequent involvement of its downstream pathways, determines different relevant physio-pathological alterations, leading the cells to acquire a greater ability to migrate, invade, proliferate and survive, fundamental mechanisms for the acquisition of resistance to common therapies. The complete understanding of these events, and the specific role of the investigated signaling pathways, could allow the correlation between tumor pathological mechanisms and the identification of future useful diagnostic and prognostic biomarkers in gliomas.

### Supplementary Information

Below is the link to the electronic supplementary material.

Supplementary file1 (PDF 2972 KB)

## Data Availability

The datasets generated and analyzed during the current study are available in the Chinese Glioma Genome Atlas (CGGA) RNA sequencing (RNA-seq) dataset (mRNAseq_325), available at the link http://www.cgga.org.cn/. All data and information related to the 50 glioblastoma patient tissue samples, used in this study, are included in the present article and in the supplementary Table 1.
