# Supplementary material for: Impact of phospholipase C β1 in glioblastoma: a study on the main mechanisms of tumor aggressiveness
Source: Cell Mol Life Sci. 2022 Mar 18;79(4):195. doi: 10.1007/s00018-022-04198-1 (PMC8933313; doi:10.1007/s00018-022-04198-1)
Supplement: Supplementary file 1 — Supplementary file1 (PDF 2972 KB) [file 18_2022_4198_MOESM1_ESM.pdf]

Supplementary Information:

Article Title: Impact of Phospholipase C β1 in Glioblastoma: a study on the main mechanisms of tumor aggressiveness

Journal Name: Cellular and Molecular Life Sciences (CMLS)

Authors: Stefano Ratti, Maria Vittoria Marvi, Sara Mongiorgi, Eric Owusu Obeng, Isabella Rusciano, Giulia Ramazzotti, Luca Morandi, Sofia Asioli, Matteo Zoli, Diego Mazzatenta, Pann-Ghill Suh, Lucia Manzoli, Lucio Cocco.

Corresponding author:

Lucio Cocco: lucio.cocco@unibo.it

Cellular Signalling Laboratory, Department of Biomedical and Neuromotor Sciences (DIBINEM), University of Bologna, Bologna 40126, Italy.

Supplementary Tables:

	MGMT	IDH1	IDH2	H3F3A	H3C2	H3C3	TERT	date of birth	age at diagnosis	Gender
Patient 1	UNMET	WT	WT	WT	WT	WT	g.1,295,113 G>A (VAF:39%)	01/02/25	68	M
Patient 2	UNMET	WT	WT	WT	WT	WT	g.1,295,113 G>A (VAF:26%)	08/09/35	57	M
Patient 3	MET	WT	WT	WT	WT	WT	g.1,295,113 G>A (VAF:42%)	23/01/29	69	M
Patient 4	MET	WT	WT	WT	WT	WT	g.1,295,113 G>A (VAF:42%)	29/05/23	69	F
Patient 5	MET	WT	WT	WT	WT	WT	WT	20/10/28	63	F
Patient 6	MET	WT	WT	WT	WT	WT	g.1,295,113 G>A (VAF:36%)	30/09/20	73	M
Patient 7	MET	WT	WT	WT	WT	WT	g.1,295,113 G>A (VAF:51%)	01/04/32	62	F
Patient 8	UNMET	WT	WT	WT	WT	WT	WT	27/10/45	54	M
Patient 9	MET	WT	WT	WT	WT	WT	g.1,295,113 G>A (VAF:44%)	22/03/34	66	M
Patient 10	UNMET	WT	WT	WT	WT	WT	WT	07/12/40	75	M
Patient 11	MET	p.R132H (VAF:44%)	WT	WT	WT	WT	WT	15/01/42	51	M
Patient 12	MET	p.R132H (VAF:30%)	WT	WT	WT	WT	WT	03/02/62	36	M
Patient 13	MET	p.R132H (VAF:34%)	WT	WT	WT	WT	WT	25/12/31	65	M
Patient 14	MET	WT	WT	WT	WT	WT	g.1,295,113 G>A (VAF:43%)	25/05/33	60	M
Patient 15	NA	WT	WT	WT	WT	WT	g.1,295,113 G>A (VAF:59%)	08/02/35	63	F
Patient 16	MET	p.R132H (VAF:43%)	WT	WT	WT	WT	WT	25/12/31	65	M
Patient 17	UNMET	WT	WT	WT	WT	WT	g.1,295,113 G>A (VAF:48%)	24/06/57	45	M
Patient 18	UNMET	WT	WT	WT	WT	p. T33T (VAF:50%)	g.1,295,113 G>A (VAF:42%)	27/06/31	72	F
Patient 19	MET	WT	WT	WT	WT	WT	g.1,295,113 G>A (VAF:55%)	28/05/29	68	F
Patient 20	NA	WT	WT	WT	WT	WT	g.1,295,113 G>A (VAF:47%)	02/03/47	56	M
Patient 21	MET	WT	WT	WT	WT	WT	g.1,295,135 G>A (VAF:41%)	02/06/37	66	M
Patient 22	UNMET	WT	WT	WT	WT	WT	g.1,295,113 G>A (VAF:38%)	23/06/32	70	M
Patient 23	UNMET	WT	WT	WT	WT	WT	g.1,295,135 G>A (VAF:64%)	07/02/61	41	M
Patient 24	UNMET	WT	WT	WT	WT	WT	g.1,295,113 G>A (VAF:37%)	08/09/32	70	M
Patient 25	UNMET	WT	WT	WT	WT	p. T33T (VAF:48%)	g.1,295,113 G>A (VAF:37%)	16/04/68	35	M
Patient 26	UNMET	p.R132H (VAF:11%)- p.W124* (VAF:5%)	WT	WT	WT	WT	g.1,295,113 G>A (VAF:55%)	12/08/44	58	M
Patient 27	UNMET	WT	WT	WT	WT	WT	g.1,295,113 G>A (VAF:47%)	05/10/33	69	M
Patient 28	UNMET	NA	WT	NA	WT	p. T33T (VAF 50%)	g.1,295,113 G>A (VAF:54%)	30/06/43	59	M
Patient 29	UNMET	WT	WT	WT	WT	WT	g.1,295,113 G>A (VAF:24%)	03/05/44	58	M
Patient 30	MET	WT	WT	WT	WT	WT	g.1,295,113 G>A (VAF:47%)	06/04/35	67	F
Patient 31	UNMET	WT	WT	WT	WT	WT	g.1,295,113 G>A (VAF:5%)	12/01/36	66	F
Patient 32	MET	WT	WT	NA	WT	WT	g.1,295,113 G>A (VAF:45%)	18/02/36	62	M
Patient 33	UNMET	WT	WT	WT	WT	WT	g.1,295,113 G>A (VAF:52%)	20/07/31	65	F
Patient 34	UNMET	WT	WT	WT	WT	WT	g.1,295,113 G>A (VAF:33%)	19/05/26	71	F
Patient 35	MET	WT	WT	WT	WT	WT	g.1,295,113 G>A (VAF:52%)	02/04/31	66	F
Patient 36	MET	p.R132H (VAF:36%)	WT	NA	WT	WT	WT	25/12/31	65	M
Patient 37	UNMET	WT	WT	WT	WT	p. T33T (55%)	g.1,295,113 G>A (VAF:31%)	25/05/45	52	M
Patient 38	UNMET	WT	WT	WT	WT	WT	g.1,295,135 G>A (VAF:18%)	19/12/29	66	M
Patient 39	MET	WT	WT	WT	WT	WT	g.1,295,113 G>A (VAF:41%)	18/02/36	60	M
Patient 40	MET	WT	WT	WT	WT	WT	g.1,295,113 G>A (VAF:44%)	12/04/25	68	M
Patient 41	MET	NA	NA	NA	NA	NA	NA	22/10/56	44	F
Patient 42	NA	NA	NA	NA	NA	NA	NA	15/02/53	46	F
Patient 43	UNMET	NA	NA	NA	NA	NA	NA	29/03/31	69	M
Patient 44	NA	NA	NA	NA	NA	NA	NA	30/08/34	67	M
Patient 45	MET	NA	NA	NA	NA	NA	NA	14/09/27	72	M
Patient 46	NA	NA	NA	NA	NA	NA	NA	25/02/33	70	M
Patient 47	MET	NA	NA	NA	NA	NA	NA	23/07/51	50	M
Patient 48	MET	NA	NA	NA	NA	NA	NA	27/06/43	57	F
Patient 49	UNMET	NA	NA	NA	NA	NA	NA	13/10/46	54	F
Patient 50	NA	NA	NA	NA	NA	NA	NA	28/02/32	67	F

Supplementary Table 1: Data and Molecular characterization of samples from 50 Glioblastoma patients

Among the 50 glioblastoma samples, only six were detected mutant for isocitrate dehydrogenase 1 (*IDH1*), *p.R132H* and none for isocitrate dehydrogenase 2 (*IDH2*) and histone *H3-3A.*These IDH1-mutated samples will be classified as Adult-type diffuse astrocytoma, IDH mutant, grade 4 considering the recent tumor classification update(1). The Telomerase Reverse Transcriptase (*TERT*) promoter was found to be mutated in 32 cases (29 for g.1,295,113 G>A and 3 for g.1,295,135 G>A). O-6-Methylguanine-DNA Methyltransferase (MGMT) was detected hypermethylated in 23 cases.

Supplementary Figures:

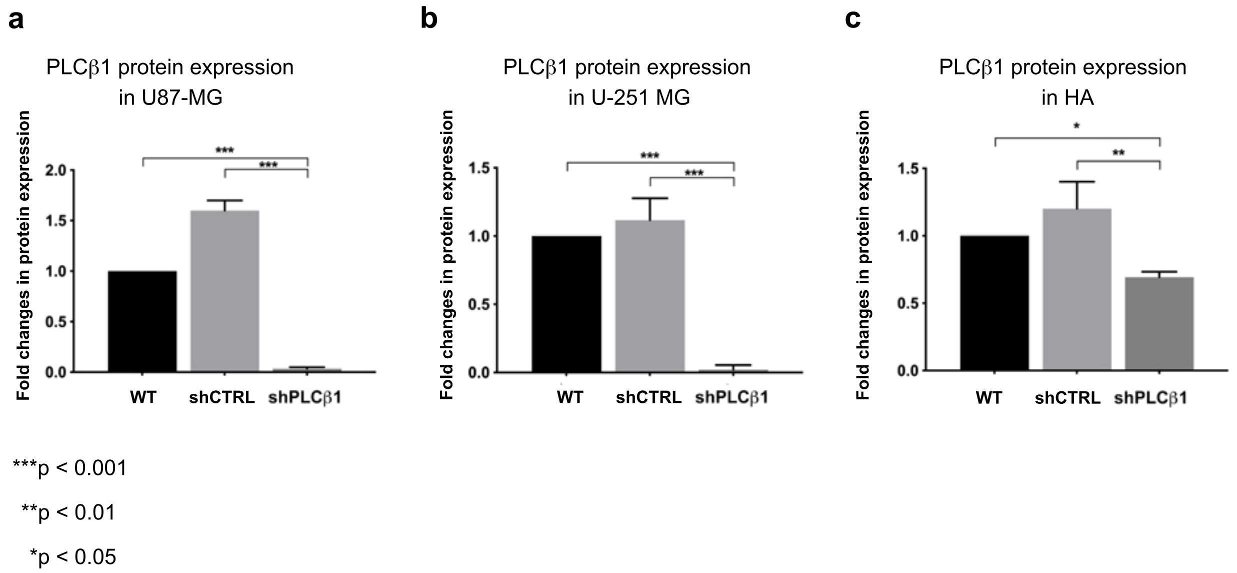

Supplementary Fig. 1: Quantitative analysis of PLCβ1 protein expression

Panels a, b and **c:** Quantitative analysis of PLCβ1 protein expression in U87-MG (**a**), U-251 MG (**b**) and HA (**c**). PLCβ1-silenced cells (shPLCβ1) were compared to wild type (WT) and mock-transduced (shCTRL) cells. WT cells were used as reference samples. Columns show the mean ± SD of three independent experiments with *p < 0.05, **p < 0.01 and ***p < 0.001.

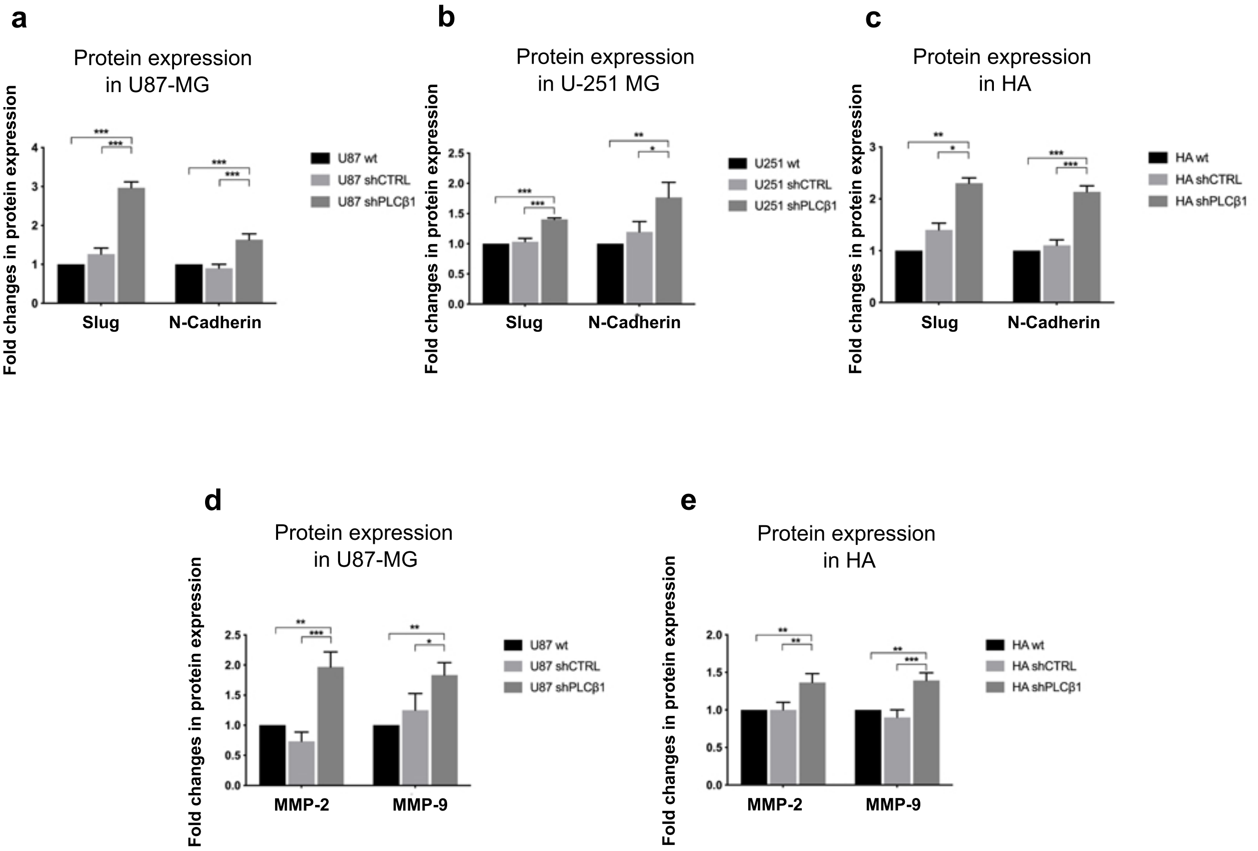

***p < 0.001
**p < 0.01
*p < 0.05

Supplementary Fig. 2: Quantitative analysis of Mesenchymal markers and MMPs protein expression

Panels a, b and **c:** Quantitative analysis of Slug and N-Cadherin protein expression in U87-MG (**a**), U-251 MG (**b**) and HA (**c**). PLCβ1-silenced cells (shPLCβ1) were compared to wild type (WT) and mock-transduced (shCTRL) cells. WT cells were used as reference samples. Columns show the mean ± SD of three independent experiments with *p < 0.05, **p < 0.01 and ***p < 0.001. **Panels d** and **e:** Quantitative analysis of MMP-2 and MMP-9 protein expression in U87-MG (**d**) and HA (**e**). PLCβ1-silenced cells (shPLCβ1) were compared to wild type (WT) and mock-transduced (shCTRL) cells. WT cells were used as reference samples. Columns show the mean ± SD of three independent experiments with *p < 0.05, **p < 0.01 and ***p < 0.001.

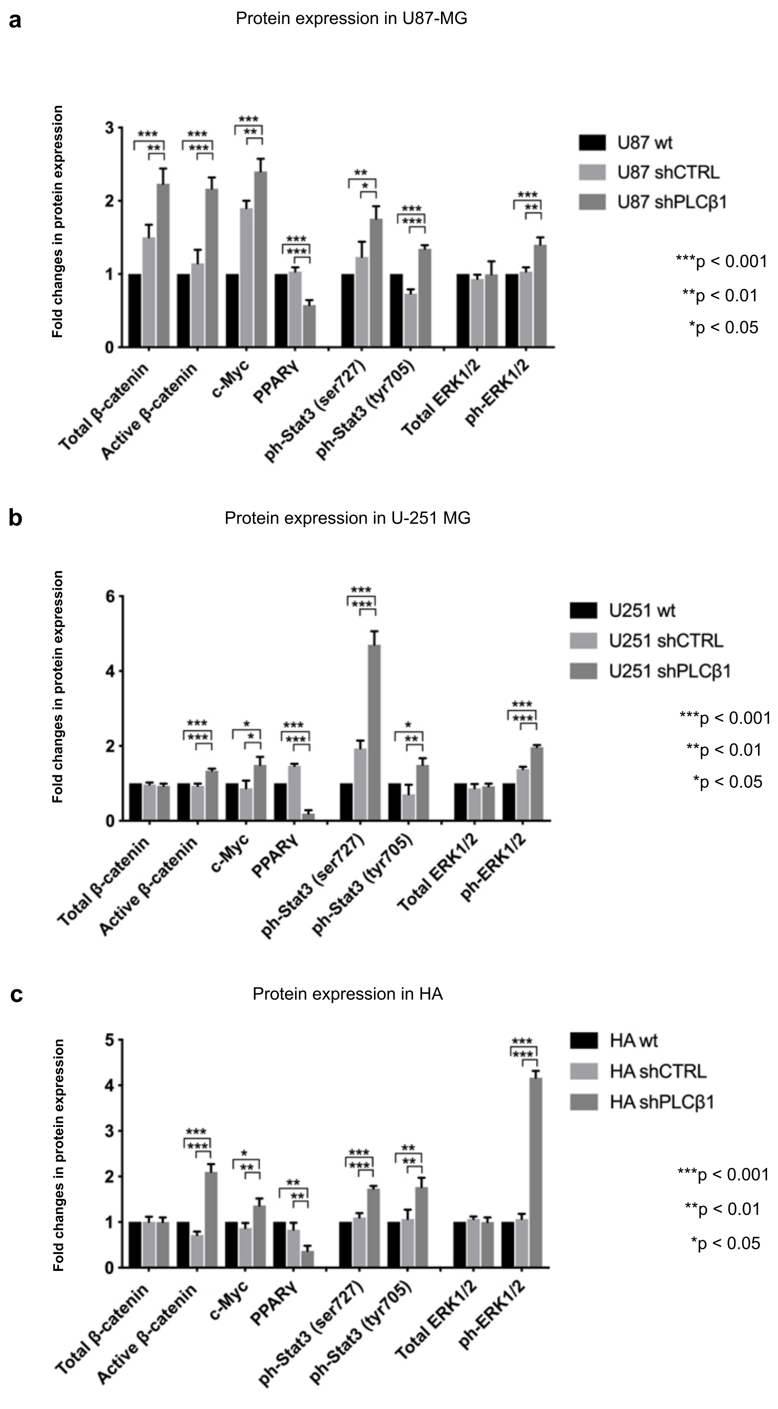

Supplementary Fig. 3: Quantitative analysis of the protein expression of the molecules belonging to the main survival pathways

Panels a, b and **c:** Quantitative analysis of the protein expression of the molecules belonging to the main survival pathways in U87-MG (**a**), U-251 MG (**b**) and HA (**c**). PLCβ1-silenced cells (shPLCβ1) were compared to wild type (WT) and mock-transduced (shCTRL) cells. WT cells were used as reference samples. Columns show the mean ± SD of three independent experiments with $*p < 0.05$, $**p < 0.01$ and $***p < 0.001$.

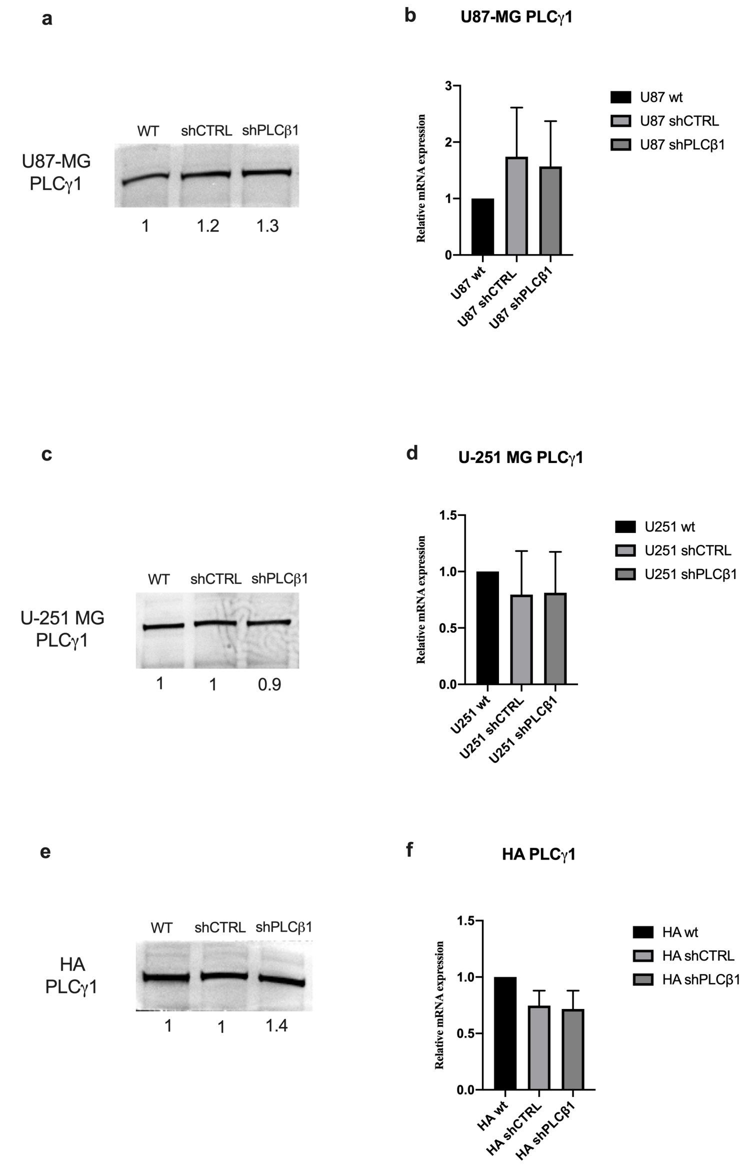

Supplementary Fig. 4: Consequences of PLCβ1 modulation on PLCγ1 expression

Panels a, c and **e:** Western blot analysis of PLCγ1 expression after PLCβ1 silencing on U87-MG (**a**), U-251 MG (**c**) and HA primary astrocytes (**e**). PLCβ1-silenced cells (shPLCβ1) were compared to wild type (WT) and mock-transduced (shCTRL) samples. Densitometric analysis was performed with total protein normalization through the iBright analysis software. **Panels b, d** and **f:** PLCγ1 mRNA expression in U87-MG (**b**), U-251 MG (**d**) and HA primary astrocytes (**f**). PLCβ1-silenced cells (shPLCβ1) were compared to wild type (WT) and mock-transduced (shCTRL) samples. GAPDH was used as housekeeping gene and all the analysis derived from three independent experiments.
